# Domain in Fiber-2 interacted with KPNA3/4 significantly affects the replication and pathogenicity of the highly pathogenic FAdV-4

**DOI:** 10.1080/21505594.2021.1888458

**Published:** 2021-02-22

**Authors:** Quan Xie, Weikang Wang, Luyuan Li, Qiuqi Kan, Hui Fu, Tuoyu Geng, Tuofan Li, Zhimin Wan, Wei Gao, Hongxia Shao, Aijian Qin, Jianqiang Ye

**Affiliations:** a Key Laboratory of Jiangsu Preventive Veterinary Medicine, Key Laboratory for Avian Preventive Medicine, Ministry of Education, College of Veterinary Medicine, Yangzhou University, Yangzhou, China; b Jiangsu Co-innovation Center for Prevention and Control of Important Animal Infectious Diseases and Zoonoses, Yangzhou, China; c Joint International Research Laboratory of Agriculture and Agri-Product Safety, the Ministry of Education of China, Yangzhou University, Yangzhou, China; d Institutes of Agricultural Science and Technology Development, Yangzhou University, Yangzhou, China; e College of Animal Science and Technology, Yangzhou University, Yangzhou, China

**Keywords:** FAdV-4, Fiber-2, KPNA3/4, interaction, gene edition, pathogenesis, live-attenuated vaccine

## Abstract

The outbreaks of hepatitis-hydropericardium syndrome (HPS) caused by the highly pathogenic serotype 4 fowl adenovirus (FAdV-4) have caused a huge economic loss to the poultry industry globally since 2013. Although the Fiber-2 has been identified as a key virulent related factor for FAdV-4, little is known about its molecular basis. In this study, we identified the efficient interaction of the Fiber-2 with the karyopherin alpha 3/4 (KPNA3/4) protein via its N-terminus of 1–40aa. The analysis of the overexpression and knockout of KPNA3/4 showed that KPNA3/4 could efficiently assist the replication of FAdV-4. Moreover, a *fiber-2*-edited virus FAV-4_Del with a deletion of 7–40aa in Fiber-2 was rescued through the CRISPR-Cas9 technique. In comparison with the wild type FAdV-4, FAV-4_Del was highly attenuated *in vitro* and *in vivo*. Notably, the inoculation of FAV-4_Del in chickens could provide full protection against the lethal challenge with the wild type FAdV-4. All these findings not only give novel insights into the molecular basis for the pathogenesis of Fiber-2 but also provide efficient targets for developing antiviral strategies and live-attenuated vaccine candidates against the highly pathogenic FAdV-4.

## Introduction

Fowl adenoviruses (FAdVs) are non-enveloped double-stranded DNA viruses, which belong to the family *Adenoviridae, aviadenovirus* [1]. FAdVs are grouped into five species (FAdV-A to FAdV-E) with 12 serotypes (FAdV-1 to 8a and 8b to 11) based on restriction enzyme digest pattern and serum cross-neutralization test [2]. Different from other serotype fowl adenoviruses, FAdV-4 mainly causes hepatitis-hydropericardium syndrome (HHS) in 3–6 week-old broiler chickens with high morbidity and mortality [3–5]. HHS caused by FAdV-4 was first reported in Pakistan in 1987 [6], and then spread into other countries and regions, including Iraq, India, Japan, Mexico, Chile, Ecuador, Poland, Russia, Slovakia, Hungary, Korea, and China [7,8], and resulted in enormous economic loss to the poultry industry worldwide. However, the molecular pathogenesis of FAdV-4 is barely known. Fibers are thought to be critical for viral infection and pathogenesis of FAdV [9]. It should be noted that serotypes FAdV-1, FAdV-4, and FAdV-10 have two Fibers (Fiber-1 and Fiber-2). Zhang et al recently reported that Fiber-2 and Hexon, but not Fiber-1, were the virulent determiners for the highly pathogenic FAdV-4 isolates in comparison with the nonpathogenic strain [10]. Our group and other group’s recent studies found that the Fiber-1, not Fiber-2, directly triggered the viral infection of FAdV-4 via its shaft and knob domains [11,12]. However, the molecular basis of the Fiber-2 in the pathogenesis of the highly pathogenic FAdV-4 need to be further elucidated. Here, Fiber-2 was identified to be interacted with the karyopherin alpha 3/4 (KPNA3/4) via its N-terminus of 1–40aa, and KPNA3/4 could facilitate the replication of FAdV-4. Moreover, a rescued FAV-4-Del virus with a deletion of 7–40aa in Fiber-2 was highly attenuated *in vitro* and *in vivo*, and could provide efficient protection against the lethal challenge.

## Results

### Fiber-2 efficiently interacted with host KPNA3/4

**Figure 1.**
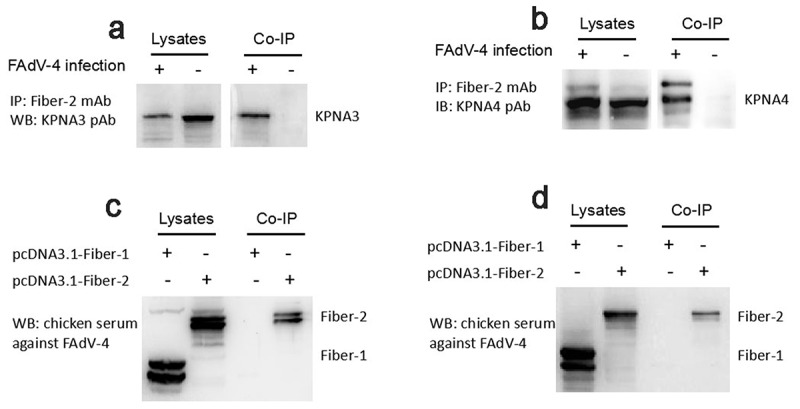
**Fiber-2 efficiently interacted with host KPNA3/4**. (a-b) LMH cells infected with FAdV-4 were immunoprecipitated with mAb 3C2 against Fiber-2. The cell lysates and the immunoprecipitates were examined with western blot using polyclonal antibodies against KPNA3 (a) and KPNA4 (b). (c-d) LMH cells transfected with pcDNA3.1-F1 or pcDNA3.1-F2 were immunoprecipitated with polyclonal antibodies against KPNA3 (c) and KPNA4 (d). The cell lysates and the immunoprecipitates were examined with western blot using chicken sera against FAdV-4. All experiments were performed for three times with comparable results

To elucidate the molecular basis of the Fiber-2 in the pathogenesis of the highly pathogenic FAdV-4, LMH cells infected with FAdV-4 for 48 hours were harvested and subjected to Co-IP with mAb 3C2 against Fiber-2. The LMH cells without infection as a control. After separated by SDS-PAGE, the immunoprecipitates were visualized by silver stain according to the manufacture’s instructions, the differential bands in the infected cells were excised from the gel and sent for mass spectrum analysis. Data from mass spectrum identified several host proteins as suspectable proteins with high reliability (unique peptide count ≥2) to interact with the Fiber-2 of FAdV-4. Among these suspectable proteins, only KPNA3 and KPNA4 proteins were denoted as characterized proteins in chicken. Moreover, KPNA3/4 plays vital roles in protein nuclear transportation and in regulating transcriptional activity, innate immune response and cell death. To confirm the interaction between KPNA3/4 and Fiber-2, the Co-IP and western blot analysis using specific antibodies against KPNA3/4 or Fiber-2 were performed. As described in Figure 1a,b, the endogenous KPNA3 and KPNA4 could be easily immunoprecipitated by mAb 3C2 against Fiber-2 in the LMH cells infected with FAdV-4, but not in the control LMH cells. Moreover, the Fiber-2 in the LMH cells transfected with pcDNA3.1-F2 could be efficiently immunoprecipitated by antibodies against either KPNA3 or KPNA4 whereas the Fiber-1 in the LMH cells transfected with pcDNA3.1-F1 could not be immunoprecipitated by antibodies against either KPNA3 or KPNA4 (Figure 1c,d). All these data clearly demonstrate that the Fiber-2 of FAdV-4 can interact with the cellular protein KPNA3/4.

### KPNA3/4 facilitates FAdV-4 replication in LMH cells

**Figure 2.**
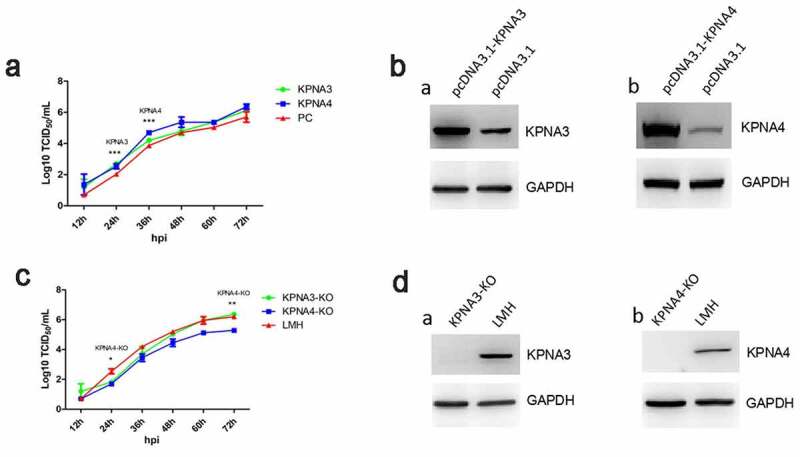
**KPNA3/4 assisted viral replication of FAdV-4 in LMH cells**. (a-b) Overexpression of KPNA3 and KPNA4 increased viral replication in LMH cells. (a) LMH cells were first transfected with pcDNA3.1-KPNA3, pcDNA3.1-KPNA4 and pcDNA3.1, respectively. 24 hours after transfection, the LMH cells were infected with FAdV-4, and the supernatants collected from infected LMH cells at indicated time points were then titrated with TCID_50_. The overexpression of KPNA3 and KPNA4 in LMH cells were examined with western blot by polyclonal antibodies against KPNA3 (b-a) and KPNA4 (b-b). (c-d) Knockout of KPNA3 or KPNA4 inhibited viral replication in LMH cells. (c) KPNA3-KO LMH cells, KPNA4-KO LMH cells and LMH cells were infected with FAdV-4, and the supernatants collected from infected LMH cells at indicated time points were then titrated with TCID_50_. The expression of KPNA3 and KPNA4 in KPNA3-KO and KPNA4-KO LMH cells were examined with western blot by polyclonal antibodies against KPNA3 (d-a) and KPNA4 (d-b), respectively. All experiments were done in triplicates and repeated twice

To investigate the role of KPNA3/4 in viral replication of FAdV-4, the KPNA3/4 genes were first amplified and cloned into the eukaryotic expression vector pcDNA3.1, designated as pcDNA3.1-KPNA3 and pcDNA3.1-KPNA4, respectively. And the LMH cells with the knockout of KPNA3 or KPNA4 were generated by using CRISPR-Cas9 technique, designated as KPNA3-KO and KPNA4-KO, respectively. The viral growth kinetics of FAdV-4 in LMH cells with overexpression or knockout of KPNA3/4 were performed. As described in Figure 2a, the over-expression of KPNA3 increased viral titer at early time points, while the over-expression of KPNA4 increased viral titer at all the time points tested. Moreover, the knockout of KPNA3 could slightly inhibit viral replication at early time points, while the knockout of KPNA4 inhibits viral replication at all the time points tested as shown in Figure 2c. Therefore, the data from the LMH cells with the knockout of KPNA3/4 were consistent with that from the LMH cells with the over-expression of KPNA3/4. The expression of KPNA3/4 in the LMH cells transfected with pcDNA3.1-KPNA3/4 or in the LMH cells with the knockout of KPNA3/4 were confirmed as described in Figure 2b,d. These data indicate that KPNA3/4 assists the viral replication of FAdV-4 in LMH cells.

### N-terminal 1-40aa in Fiber 2 response for the interaction with KPNA3/4

**Figure 3.**
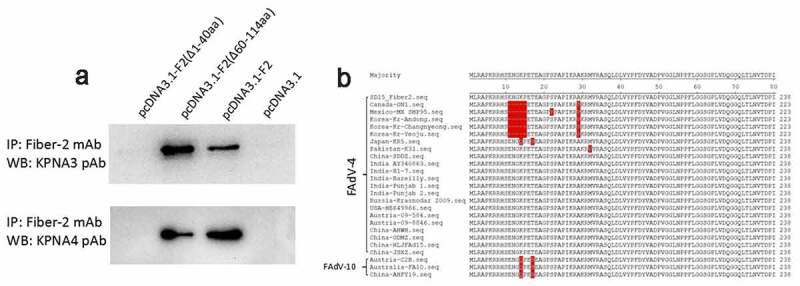
**N-terminal 1–40aa in Fiber 2 responsed for the interaction with KPNA3/4**. LMH cells were transfected with full-length *fiber-2*, truncated *fiber-2* (1–40 aa deletion), truncated *fiber-2* (61–114 aa deletion) and pcDNA3.1, respectively. (a) Cell lysates were prepared and immunoprecipitated with mAb 3C2 against Fiber-2, the pellets were examined with western blot by polyclonal antibodies against KPNA3 and KPNA4. (b) Sequence alignment analysis for N-terminus of 1–40aa in Fiber-2 from different FAdV-4 isolates. All experiments were performed for three times with comparable results

To map the domain in Fiber-2 responsible for the interaction with KPNA3/4, the plasmids carrying the different truncated *fiber-2* were constructed. After a series of Co-IP and western blot analysis for these Fiber-2 truncations, N-terminal 1–40aa in Fiber-2 was identified to be responsed for the interaction with KPNA3/4. As described in Figure 3a, the endogenous KPNA3/4 could be efficiently immunoprecipitated by mAb 3C2 in LMH cells transfected with full-length *fiber-2* and the truncated *fiber-2* with 61–114aa deletion, but not in LMH cells transfected with the truncated *fiber-2* with 1–40aa deletion. Notably, large sequences analysis revealed that N-terminal 1–40aa in Fiber-2 was highly conserved in the highly pathogenic FAdV-4 isolates, but several unique deletions or mutations were found in the nonpathogenic FAdV-4 isolates (Figure 3b). Several mutations were also found in the nonpathogenic FAdV-10 serotype. These findings indicate that the N-terminal 1–40aa in Fiber-2 interacted with KPNA3/4 might play a key role in the pathogenesis of FAdV-4.

### Generation of FAV4_Del carrying Fiber-2 without N-terminal 7-40aa

**Figure 4.**
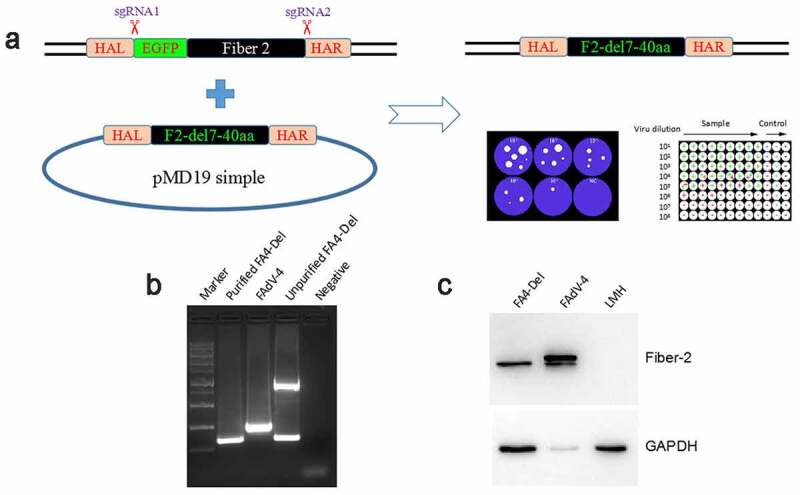
**Generation of FAV4_Del carrying Fiber-2 without N-terminal 7–40aa**. (a) Strategy of the CRISPR/Cas9 platform for generating the *fiber-2*-edited virus FAV4_Del. LMH cells were first transfected with sgRNA1 and sgRNA2, and then LMH cells were infected with FAdV-4-EGFP and transfected with donor plasmid at 24 hours post-transfection. The *fiber-2*-edited virus FAV4_Del was then purified by limiting dilution assay and viral plaque assay. (b) PCR identification of the *fiber-2*-edited virus FAV4_Del. Viral genome of purified FAV4_Del, FAdV-4 and unpurified FAV4_Del were extracted and identified by PCR. (c) Western blot analysis of the *fiber-2*-edited virus FAV4_Del. LMH cells were infected with the *fiber-2*-edited virus FAV4_Del and FAdV-4. 4 d post-infection, the infected LMH cells were harvested and lysed, and the lysates were then examined with western blot by mAb 1C9 against Fiber-2. Experiments B and C were performed for three times with comparable results

**Figure 5.**
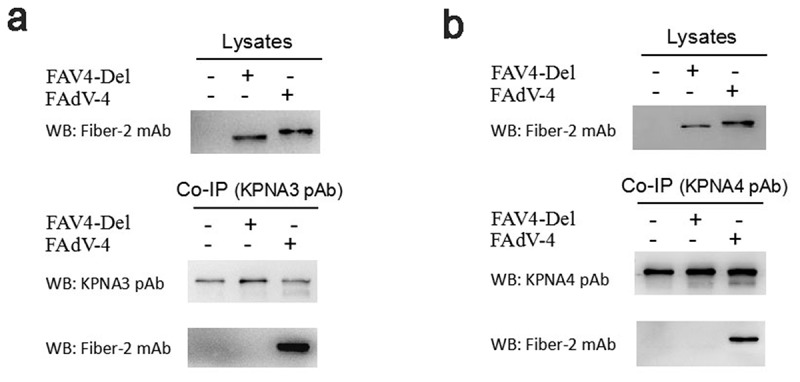
**Evaluation of the interaction between KPNA3/4 and FAV4-Del**. LMH cells were infected with FAdV-4 and FAV4-Del at 0.1 MOI respectively, and lysed and immunoprecipitated with KPNA3 (a) or KPNA4 (b) polyclonal antibodies, and then the cell lysates were detected with mAb 1C9 against Fiber-2, while the immunoprecipitates were detected with polyclonal antibodies against KPNA3(A) and KPNA4 (B), and mAb 1C9 against Fiber-2, respectively

To elucidate the roles of the N-terminal 1–40aa in Fiber-2 interacted with KPNA3/4 in viral replication and pathogenesis of FAdV-4, a *fiber-2*-edited FAdV-4 virus-carrying Fiber-2 without N-terminal 7–40aa, designated as FAV4_Del, was rescued by the co-transfection of two sgRNAs targeting the EGFP and *fiber-2*, and the donor plasmid into LMH cells followed by the infection of FAdV4-EGFP as shown in Figure 4a. The rescued FAV4_Del was then purified by several limiting dilution and plaque assay. The purified FAV4_Del was confirmed by PCR, sequencing and western blot. As described in Figure 4b, the single and specific band for FAV4_Del could be amplified from the purified FAV4_Del whereas two bands (the small band for FAV4_Del and the large band for FAdV4-EGFP) could be amplified in the unpurified FAV4_Del. Sequencing further confirmed that the *fiber-2*-edited FAV4_Del was exactly the same as designed (Data not shown) which carrying the deletion of N-terminal 7–40aa in its Fiber-2. Western blot assay also showed that the molecular weight of Fiber-2 expressed in LMH cells infected with FAV4_Del was slightly smaller than that of the full-length Fiber-2 expressed in LMH cells infected with the wild type FAdV-4 (Figure 4c). All these data clearly demonstrate that the *fiber-2*-edited FAV4_Del with the deletion of 7–40aa in Fiber-2 is efficiently generated and purified. To further evaluate whether the KPNA3/4 could interact with FAV4-Del through the truncated Fiber-2, LMH cells infected with FAdV-4 and FAV4-Del, respectively, were lysed and immunoprecipitated by antibodies against KPNA3 or KPNA4, respectively, and then detected with mAb 1 C9 against Fiber-2. As shown in Figure 5, only the Fiber-2 in the cells infected with FAdV-4, but not in cells infected with FAV4-Del could be efficiently immunoprecipitated by antibodies against KPNA3 or KPNA4, highlighting that KPNA3/4 can not interact with the *fiber-2*-edited FAV4-Del through the truncated Fiber-2.

### FAV4_Del showed low replication ability in LMH cells

**Figure 6.**
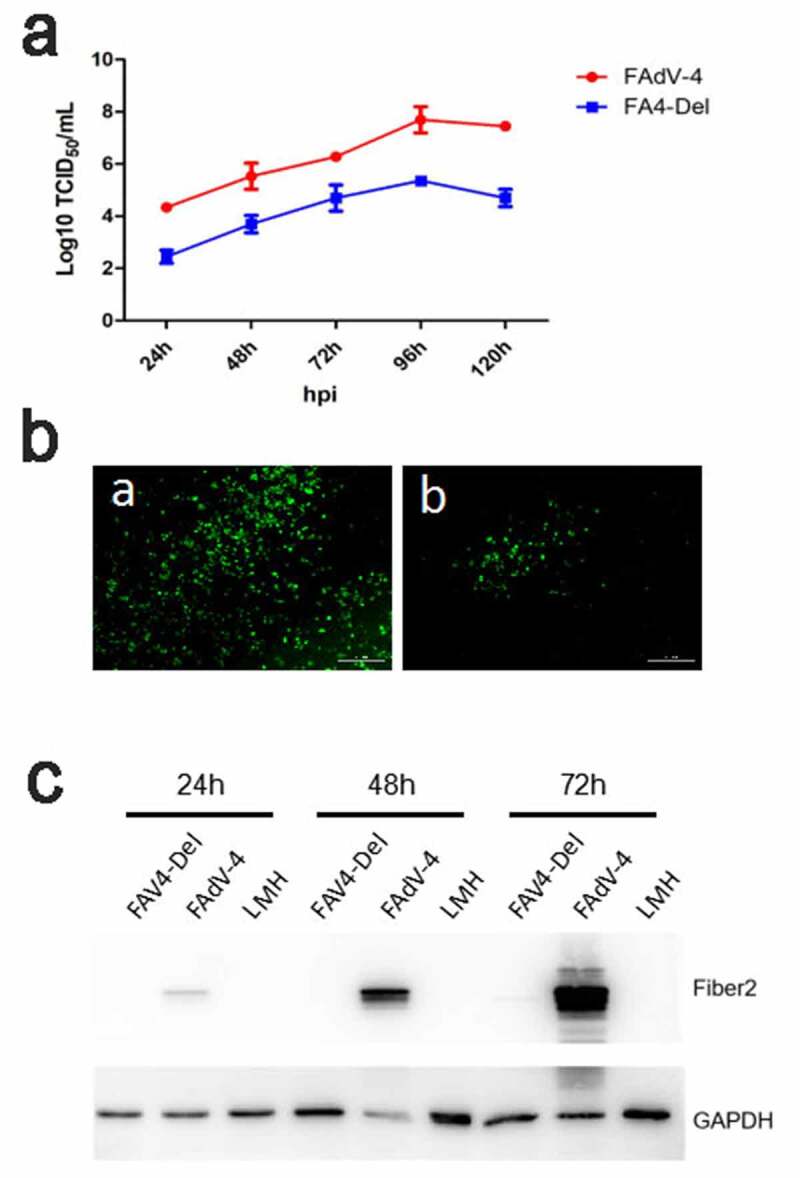
**FAV4_Del showed low replication ability in LMH cells**. (a) LMH cells were infected with FAV4_Del and FAdV-4 at the same dose, the viral supernatant collected from infected LMH cells at indicated time points were then titrated with TCID_50_. (b) LMH cells infected with the FAV4_Del and FAdV-4 were analyzed by IFA using mAb 3B5 against Fiber-1. (c) LMH cells infected with FAV4_Del and FAdV-4 were harvested at different time points and lysed by lysis buffer, and then the lysates were examined with western blot by mAb 1C9 against Fiber-2. All experiments were performed for three times with comparable results

To test the viral replication ability of the rescued FAV4_Del, the LMH cells were infected with FAV4_Del and wild type FAdV-4 at 0.01 MOI, respectively, and the viral growth kinetics of the two viruses in the LMH cells were compared. As shown in Figure 6a, the FAV4_Del replicated significantly slower than the wild-type FAdV-4. The viral titer of FAV4_Del was about 100 times lower than that of the wild type FAdV-4 at 24 hpi-120 hpi. Notably, the peak titer of FAdV-4 could reach to 6 × 10^7^ TCID_50_/ml whereas that of FAV4_Del was only 10^5^ TCID_50_/ml at 96 hpi. This data was confirmed by IFA and western blot analysis. As described in Figure 6b, a lot of specific immunofluorescences could be found in the LMH cells infected with FAdV-4 but only a few immunofluorescences could be detected in the LMH cells infected with FAV4_Del at 96 hpi. In the western blot, the Fiber-2 protein in the LMH cells infected with FAdV-4 could be easily detected at 24 hpi, 48 hpi and 72 hpi whereas that in the LMH cells infected with FAV4_Del was barely detectable (Figure 6c). All these data demonstrate that the N-terminal 7–40aa of Fiber-2 significantly affect the viral replication of FAdV-4 in LMH cells.

### FAV4_Del was highly attenuated in vivo

**Figure 7.**
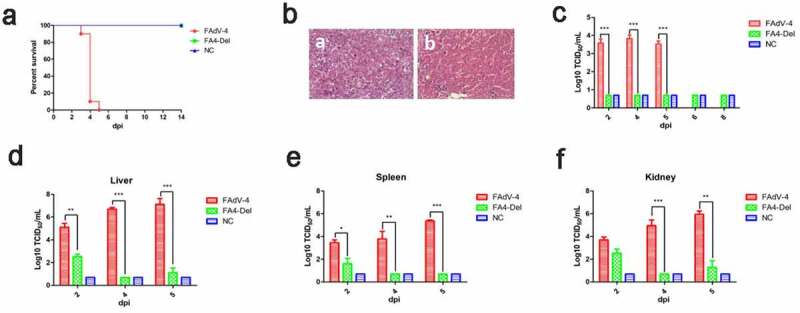
**FAV4_Del was highly attenuated *in vivo.*** All the SPF chickens were randomly divided into three groups, and inoculated with FAV4_Del, wild type FAdV-4 and 1% culture medium (NC) respectively. All the SPF chickens were observed for 14 d. (a) Percent of survival for the infected chickens. (b) Representative histological changes in liver tissues from chickens infected with FAdV-4 (a) and FAV4_Del (b). (c) Viral shedding in cloacal swabs from the infected chickens. Viral loads in liver (d), spleen (e) and kidney (f) tissues from the infected chickens

To evaluate the viral replication and pathogenesis of FAV4_Del *in vivo*, SPF chickens were infected with FAV4_Del and wild-type FAdV-4 at the same dose. The clinical symptoms and mortality of the infected chickens were monitored daily, and the liver, spleen, kidney and the cloacal swabs were collected for viral titration at different time points post-infection. At 2 dpi, the chickens infected with FAdV-4 began to show clinical signs characterized by depression, sleepiness, and huddling together with ruffled feathers. As described in Figure 7a, 10%, 90% and 100% of chickens infected with FAdV-4 were died at 3 dpi, 4 dpi and 5 dpi, respectively. However, all the chickens infected with FAV4_Del did not show any clinical signs and death throughout the experiment (Figure 7a). Necropsy analysis demonstrated that the chickens in FAdV-4 group showed hydropericardium, hepatitis with hemorrhage, kidney and spleen swelling, and hemorrhage whereas no gross lesion in heart, liver, spleen, or kidney were observed in the chickens infected with FAV4_Del (Data not shown). The HE staining further demonstrated that the degeneration and necrosis of hepatocytes, and the intranuclear inclusion bodies in hepatocytes were observed in the chickens infected with FAdV-4 whereas no histopathological symptoms were found in the chickens infected with FAV4_Del (Figure 7b). For virus shedding, the viral titers in cloacal swabs from the chickens infected with FAdV-4 could reach to 10^3^–10^4^ TCID_50_/ml at 2 dpi-5 dpi but the virus could not be detectable in the cloacal swab samples in the chickens infected with FAV4_Del at 2 dpi-8 dpi (Figure 7c). For virus loading in tissues, the viral titers in liver from the chickens infected with FAdV-4 could reach to 10^5^–10^7^ TCID_50_/ml at 2 dpi-5 dpi whereas those in the chickens infected with FAV4_Del were less than 10^3^ TCID_50_/ml at 2 dpi-5 dpi as described in Figure 7d. Notably, very similar data were found in the kidney and spleen tissues as shown in Figure 7e,f. Therefore, the data from the virus cloacal shedding and tissue loading were consistent with the data for the clinical signs and survival. All these data demonstrate that the *fiber-2*-edited FAV4_Del virus was highly attenuated in chickens.

### FAV4_Del provided efficient protection against lethal challenge

**Figure 8.**
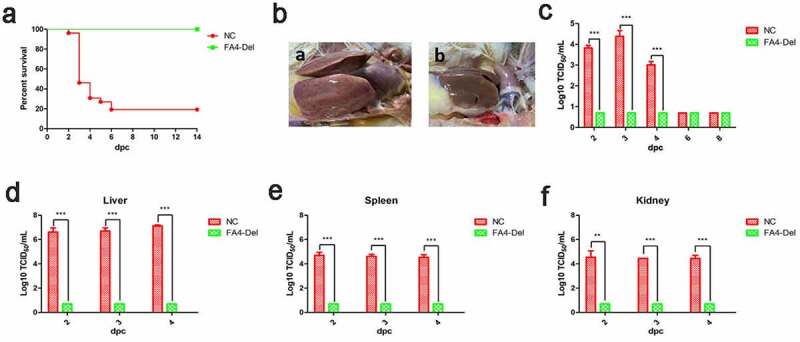
**FAV4_Del provided efficient protection against lethal challenge**. The chickens inoculated with FAV4_Del and 1% culture medium survived after 21 d were then challenged with the lethal dose of FAdV-4. (a) Percent of survival for the challenged chickens. (b) Representative gross lesion in heart and liver from the challenged control chickens (a) and the challenged chickens previously inoculated with FAV4_Del (b). (c) Viral shedding in cloacal swabs from the challenged chickens. Viral loads in liver (d), spleen (e) and kidney (f) tissues from the challenged chickens

Since FAV4_Del was highly attenuated in chickens, to further evaluate whether the FAV4_Del could be as a live-attenuated vaccine candidate, the chickens infected with FAV4_Del or the control chickens at 21 dpi were challenged with the lethal dose of the wild type FAdV-4. The clinical signs and mortality of the challenged chickens were monitored daily, and the liver, spleen, kidney, and the cloacal swabs were collected for viral titration at different time points post-challenge. After challenge, the chickens in control group exhibited clinical signs including depression, loss of appetite, and huddling together with ruffled feathers, and the mortality of these chickens reached 80% (21/26) at day 6 post-challenge (Figure 8a). In contrast, the challenged chickens previously infected with FAV4_Del showed no clinical symptoms and death throughout the experiment (Figure 8a). After necropsy, typical symptoms such as hydropericardium, hepatitis, and nephritis could be easily found in the control challenged chickens, but not in the challenged chickens previously infected with FAV4_Del (Figure 8b). Notably, these data were consistent with the viral shedding in the cloacal swabs and viral titers in tissue samples. As described in Figure 8c, high viral titers (10^3^–10^4^ TCID_50_/ml) in cloacal swabs from the challenged control group could be detected at 2 dpc (post-challenge), 3 dpc and 4 dpc whereas those no viruses were detected at all the time points in the challenged chickens previously infected with FAV4_Del (Figure 8c). Similarly, the virus loading in tissues from the challenged control group showed the highest titer in liver, followed with spleen and kidney at the similar level at 2 dpc, 3 dpc and 4 dpc whereas no viruses could be detected in the tissue samples tested from the challenged chickens previously infected with FAV4_Del at any time points. These data demonstrate that the *fiber-2*-edited FAV4_Del virus can be used as a live-attenuated vaccine candidate to provide protection against the lethal challenge of FAdV-4.

## Discussion

FAdV-4 is the causative agent for the disease of hepatitis-hydropericardium syndrome (HHS) in chickens. Since its first report in 1987, HHS caused by FAdV-4 has spread globally [6]. Recently, the outbreak of highly pathogenic FAdV-4 and its co-infection with other viral pathogens in China significantly affect the sustaining development of poultry industry. However, little is known about the molecular basis for the infection and pathogenesis of FAdV-4. Recently, our group and other group both revealed that Fiber-1, but not Fiber-2, directly triggered the viral infection of FAdV-4 via its shaft and knob domains [11,12]. Zhang et al. reported that Fiber-2, but not Fiber-1, was identified as one of the major virulent determiners for the highly pathogenic FAdV-4 endemic in China [10]. In Zhang’s studies, it should be noted that Zhang et al. used the same viral backbone to exchange Fiber-2 or Fiber-1 from the highly pathogenic FAdV-4 with that from the nonpathogenic FAdV-4 to determine the role of Fiber-2 in the highly pathogenic FAdV-4 [10]. The domain in Fiber-2 or any potential related host factor which determines the virulence of Fiber-2 is not elucidated. In this study, we revealed that Fiber-2 could efficiently interact with host KPNA3/4 and KPNA3/4 could assist the replication of FAdV-4. Except for KPNA3 and KPNA4, several other suspectable and uncharacterized proteins in chicken (Data not shown) with reliability to interact with the Fiber-2 of FAdV-4 were also found in the data from mass spectrum. The interaction between these uncharacterized proteins in chicken and Fiber-2 of FAdV-4, and their potential roles in the pathogenesis of FAdV-4 need to be further investigated.

KPNA3 and KPNA4, both belonged to the karyopherin alpha two subfamily [13,14], play vital roles in protein nuclear transportation [15–18], nuclear pore complexes (NPC) assembly [19–21], and in regulating transcriptional activity [22,23], innate immune response [24–26] and cell death [27–30]. Previous studies also demonstrated that some viruses such as Japanese encephalitis virus [31], porcine circovirus type 2 [32], Middle East respiratory syndrome coronavirus [33] and influenza virus [34–37] could hijack KPNA3/4 to competitively block the interaction of KPNA3/4 with their cargos, and then to restrict their function to establish infection or evade the host antiviral response. Our data showed that the over-expression or knockout of KPNA3/4 could decrease or increase the viral replication of FAdV-4, respectively. However, the effect of KPNA3/4 on the viral replication of FAdV-4 in our studies was not very significant. This might be related with the potential complement activity for KPNA3 and KPNA4 due to their similar functions [38]. To overcome this, we also tried to generate LMH cells with double knockout of KPNA3 and KPNA4. However, such LMH cells with double knockout of KPNA3 and KPNA4 could not be generated, indicating the key roles of KPNA3 and KPNA4 in cell survival. Recently, one study reported that KPNA3, KPNA4, and KPNB1 were important regulators of inflammation via activation of RelA/p65 and permeability via loss of cell surface VE-Cadherin in endothelial cell (EC) [39]. Considering that the infection of FAdV-4 causes a significant decrease of colloid osmotic pressure [40], it is not difficult to speculate that the interaction between Fiber-2 of FAdV-4 with KPNA3/4 might be related with hydropericardium symptom.

Except for the finding of the interaction between Fiber-2 and KPNA3/4, the N-terminal 1–40aa of Fiber-2 was identified as the binding domain in Fiber-2 with KPNA3/4. Interestingly, several unique deletions or mutations were found in the sequence of N-terminal 1–40aa of Fiber-2 in some nonpathogenic FAdV-4 isolates. For example, nonpathogenic FAdV-4 isolate ON1 has a deletion of “10ENGKP16” and A29P mutation, and isolate KR5 carries K13Q and T16S mutations as described in Figure 3b. Notably, nonpathogenic serotype 10 fowl adenovirus (FAdV-10) which belongs to the same species C with FAdV-4, also carries K13Q and T16S mutations. The roles of the deletion or these mutations in the pathogenesis of FAdV-4 need to be further elucidated.

To evaluate the contribution of the binding domain in Fiber-2 with KPNA3/4 to the pathogenesis, a *fiber-2*-edited FAV-4_Del virus with the deletion of 7–40aa of Fiber-2 was generated through CRISPR-Cas9 technique. *In vitro* and *in vivo* studies both demonstrate that N-terminal 7–40aa of Fiber-2 play vital roles in the viral replication and pathogenesis. FAV4_Del replicated significantly slower than the wild-type FAdV-4 in LMH cells, and did not shed viruses in cloaca in the infected chickens. The infection of FAdV-4 caused 100% mortality of the infected chickens whereas the infection of FAV4_Del did not cause any clinical signs and all the chickens infected with FAV4_Del were survival. Moreover, FAV4_Del can provide efficient protection in chickens against lethal challenge with the wild type FAdV-4. Again, the challenged chickens previously infected with FAV4_Del did not cause any clinical signs and all the chickens were survival whereas the challenged control chickens showed severe clinical signs with 80% mortality.

In summary, this is the first demonstration of the interaction of the Fiber-2 of FAdV-4 with KPNA3/4 via its N-terminal 1–40aa, and the generation of a novel highly attenuated *fiber-2*-edited fowl adenovirus FAV4_Del. These findings provide novel insights into the molecular basis for the pathogenesis of Fiber-2, and give potential targets for developing antiviral strategies and live-attenuated vaccine candidates against the highly pathogenic FAdV-4. However, the exact roles of KPNA3/4 in the hydropericardium symptom caused by FAdV-4, and the attenuated and protective mechanism for FAV4_Del need to be further elucidated.

## Materials and methods

### Cells, viruses, and antibodies

Leghorn male hepatoma (LMH) cells were purchased from ATCC. The KPNA3-KO, KPNA4-KO LMH cells were generated in this study and stored in our laboratory. All these cells were cultured in Dulbecco’s Modified Eagle’s Medium/F12 (Gibco, NY, USA) supplemented with 10% fetal bovine serum (Lonsera, Shanghai, China) in 5% CO_2_ incubator at 37°C. The FAdV-4 strain SD was isolated and stored in our laboratory, and propagated in LMH cells. The recombinant virus FAdV-4-EGFP was generated and stored in our laboratory. KPNA3 (ab6038) and KPNA4 (ab6039) polyclonal antibody were purchased from Abcam. Monoclonal antibody (mAb) 3B5 against Fiber-1 and mAb 3C2 against Fiber-2 were generated in our laboratory, and mAb 1C9 against Fiber-2 was kindly provided by Professor Hongjun Chen.

### Plasmid construction

pcDNA3.1-KPNA3, pcDNA3.1-KPNA4, and different *fiber-2* truncates including pcDNA3.1-F2-del_1-40aa and pcDNA3.1-F2-del_61-114aa were constructed by a CloneExpress II One Step Cloning Kit (Vazyme biotech, Nanjing, China). The primers used for these constructors were listed in Table 1. pcDNA3.1-F1 and pcDNA3.1-F2 were stored in our laboratory. The sgRNAs targeting the FAdV-4 *fiber-2*, and the KPNA3 and KPNA4 genes were designed using CRISPR guide RNA designing website (www.benchling.com) and cloned into the CRISPR/Cas9 vector lentiCRISPR v2. The sequences of the sgRNAs were listed in Table 2. The donor plasmid with homologous arm (HA) at both ends flanking the truncated *fiber-2* was constructed by PCR, then assembled as HAL-F2-HAR, and finally cloned into the pMD19 simple vector.

**Table 1.** PCR primers for plasmids construction

PCR products	Sequences of primers (5ʹ-3ʹ)
Linearized pcDNA3.1	F: GAATTCTGCAGATATCCAGCACAGTG
	R: GCTCGGTACCAAGCTTAAGTTTAAACG
pcDNA3.1-KPNA3	F: AGCTTGGTACCGAGCATGGCCGAGAACGCCGCCGCCG
	R: ATATCTGCAGAATTCTTAAAAATTAAATTCTTTTGTTTG
pcDNA3.1-KPNA4	F: AGCTTGGTACCGAGCATGGCCGACAACGAGAAACTGG
	R: ATATCTGCAGAATTCCTAAAACTGGAACCCTTCTGC
pcDNA3.1-fiber-2 (Δ1-40aa)	F: AGCTTGGTACCGAGCATGGTTTATCCTTTCGATTACGTG
	R: ATATCTGCAGAATTCTTACGGGAGGGAGGCCGC
pcDNA3.1-fiber 2 (Δ61-114aa)	F: TTGGGAATCACCCCCGATGGACTGG
	R: TCGGGGGTGATTCCCAAAAAAGGCGGGTTG

**Table 2.** Primers used for sgRNA cloning

Targeting site	Sequences of primers (5ʹ-3ʹ)
*egfp*	F: CACCGGGGCGAGGAGCTGTTCACCG
	R: AAACCGGTGAACAGCTCCTCGCCCC
*fiber-2*	F: CACCGCGTGCTCTACAGCTGTCCAG
	R: AAACCTGGACAGCTGTAGAGCACGC
KPNA3	F:CACCGATGGCCGAGAACGCCGCCGC
	R: AAACGCGGCGGCGTTCTCGGCCATC
KPNA4	F: CACCGGTTGTCCAGTTTCTCGTTGT
	R: AAACACAACGAGAAACTGGACAACC

### Mass spectrum analysis

LMH cells infected with FAdV-4 were harvested, washed, and lysed with lysis buffer (CST, MA, USA) on ice with PMSF (Beyotime, Shanghai, China), protease and phosphatase inhibitors (CST, MA, USA), the cell lysates were then incubated with mAb 3C2 against Fiber-2 for co-immunoprecipitation (Co-IP). The protein precipitated were further separated by SDS-PAGE and visualized by silver stain according to the manufacture’s instructions, the differential bands were excised from the gel and transferred to Shanghai Applied Protein Technology Company for mass spectrum analysis.

### Co-immunoprecipitation

LMH cells infected with FAdV-4 or FAV4_Del or transfected with pcDNA3.1-F1 or pcDNA3.1-F2 plasmid were harvested and lysed on ice in lysis buffer with PMSF, protease, and phosphatase inhibitors, then the cell lysate was centrifuged with 12,000 rpm at 4°C for 15 minutes. And then the lysate was mixed with 2 μg of antibodies against Fiber-2 or KPNA3/4 at 4°C overnight. Then, 30 μL of protein G-Sepharose beads were added to the mixture for additional of 3 h incubation, the mixture of protein and beads was then washed five times with PBS, boiled with protein loading buffer, followed with western blot analysis.

### Western blot assay

The LMH cells transfected with plasmids or infected with FAdV were collected and lysed in lysis buffer with PMSF, protease, and phosphatase inhibitors. The lysates were boiled in the loading buffer, and was then subjected to 10% SDS-PAGE and transferred to nitrocellulose (NC) membranes (GE Healthcare Life sciences, Freiburg, Germany). After blocked with 5% skim milk in PBST for 2 h at room temperature (RT), the membranes were reacted with the corresponding antibodies at 4°C overnight. After being washed with PBST for three times, the membrane was incubated with HRP-labeled secondary antibodies for 1 h at RT. After another three washes, the membranes were developed with chemiluminescent reagents and imaged with an automatic imaging system (Tanon 5200).

### Indirect immunofluorescent assay

The LMH cells infected with viruses were fixed with pre-chilled acetone: ethanol (3:2 v/v) mixture for 5 minutes at RT and washed with PBS. The cells were stained with the diluted mAb 3B5 against Fiber-1 for 45 min at 37°C. After washed three times with PBS, the cells were stained with the diluted second antibody (goat anti-mouse IgG-FITC) for another 45 min at 37**°C**. Again, after three washes with PBS, the cells were observed by invert fluorescence microscopy.

### Generation of the cell lines with the knockout of KPNA3 or KPNA4

LMH cells seeded in the 6-well plate were transfected with sgRNAs targeting the KPNA3 or KPNA4 genes. 48 h post-transfection, 6 μM of puromycin was added to the transfected LMH cells. The LMH cells without sgRNA cassettes were then killed within 3–5 d. KPNA3-KO or KPNA4-KO LMH cells were then isolated by serial dilution, followed by an expansion period to establish a new clonal cell line.

### Generation of the FA4_Del with the deletion of 7-40aa in the Fiber-2

sgRNAs targeting the *egfp* and *fiber-2* gene, and the donor plasmid, with each 2 μg, were co-transfected into LMH cells. 24 h post-transfection, the LMH cells were infected with FAdV-4-EGFP at 0.1 MOI. The rescued *fiber-2*-edited FAdV-4, designated as FAdV-4_Fiber-2_del7-40aa (FAV4_Del), was then purified through limiting dilution assays and virus plaque assays. The purified FAv4_Del was further identified by western blot, PCR and sequencing. Moreover, Co-IP was also carried out to evaluate the interaction between KPNA3/4 and FAV4-Del.

### Viral growth kinetics

LMH cells transfected with KPNA3 or KPNA4, and KPNA3-KO or KPNA4-KO LMH cells were infected with FAdV-4 or FA4_Del at 0.01 MOI, respectively. And then the viruses were harvested at indicated time points post-infection and stored at −80°C before use. The TCID_50_ of the harvested viruses was determined in 96-well plates by serially dilution from 10^−1^ to 10^−8^, and calculated by Reed-Muench method.

## Animal studies

A total of 120 one-day-old SPF chickens were randomly divided into three groups (40 chickens per group; Group I: chickens infected with FAdV-4; Group II: chickens infected FA4_Del; Group III: chickens inoculated with 1% culture medium). The chickens were housed in different negative-pressure isolators for 14 d, and subsequently infected with 2.5 × 10^4^ TCID_50_ of indicated virus in 200 μL 1% culture medium intramuscularly. The liver, spleen, kidney and the cloacal swabs were collected at indicated time points post-infection for viral titration or histopathology analysis. At 21 dpi, chickens survived in group II and group III were challenged with 10^6^ TCID_50_ of FAdV-4 in 200 μL 1% culture medium intramuscularly. The liver, spleen, kidney, and the cloacal swabs were collected at indicated time points post-challenge for viral titration. The clinical symptoms and mortality of the infected or challenged chickens were monitored daily.

## Quantitation of viral titers in organs and cloacal swabs

The liver, spleen, and kidney collected were homogenized, treated with 10× penicillin and streptomycin for 1 h and centrifuged to obtain the supernatant. The cloacal swabs collected from the chickens were placed in 800 μL of PBS. After three times of freeze-thaw cycles, the samples were treated the same with organs homogenates. The virus-containing supernatants were then serially diluted and inoculated into LMH cells. Four days post-infection, the infected LMH cells were fixed and detected by IFA using 3B5 mAb against Fiber-1, and the TCID_50_ of these supernatants were determined by the Reed-Muench method.

## Statistical analysis

All the results are presented as means ± standard deviation. The statistical analysis in this study was performed with a Student’s test or One-way ANOVA test using GraphPad 5 software. P-value of <0.05 was considered significant. *, **, and *** indicate P value less than 0.05, 0.01 and 0.001, respectively.

## Data Availability

The datasets used and/or analyzed during the current study are available from the corresponding author on reasonable request.
